# Acute and chronic stress alter behavioral laterality in dogs

**DOI:** 10.1038/s41598-023-31213-7

**Published:** 2023-03-11

**Authors:** Yasemin Salgirli Demirbas, Sevim Isparta, Begum Saral, Nevra Keskin Yılmaz, Deniz Adıay, Hiroshi Matsui, Gülşen Töre-Yargın, Saad Adam Musa, Durmus Atilgan, Hakan Öztürk, Bengi Cinar Kul, C. Etkin Şafak, Sebastian Ocklenburg, Onur Güntürkün

**Affiliations:** 1 Department of Physiology, Faculty of Veterinary Medicine, Ankara University, Ankara, Turkey; 2 Biopsychology, Department of Psychology, Institute of Cognitive Neuroscience, Ruhr-University Bochum, Bochum, Germany; 3 Department of Genetics, Faculty of Veterinary Medicine, Ankara University, Ankara, Turkey; 4 Department of Internal Medicine, Faculty of Veterinary Medicine, Ankara University, Ankara, Turkey; 5 Center for Human Nature, Artificial Intelligence, and Neuroscience, Hokkaido University, Hokkaido, Japan; 6 Department of Industrial Design, Middle East Technical University, Ankara, Turkey; 7 Department of Psychology, Medical School Hamburg, Hamburg, Germany; 8 ICAN Institute for Cognitive and Affective Neuroscience, Medical School Hamburg, Hamburg, Germany

**Keywords:** Cognitive neuroscience, Stress and resilience, Neurophysiology

## Abstract

Dogs are one of the key animal species in investigating the biological mechanisms of behavioral laterality. Cerebral asymmetries are assumed to be influenced by stress, but this subject has not yet been studied in dogs. This study aims to investigate the effect of stress on laterality in dogs by using two different motor laterality tests: the Kong™ Test and a Food-Reaching Test (FRT). Motor laterality of chronically stressed (n = 28) and emotionally/physically healthy dogs (n = 32) were determined in two different environments, i.e., a home environment and a stressful open field test (OFT) environment. Physiological parameters including salivary cortisol, respiratory rate, and heart rate were measured for each dog, under both conditions. Cortisol results showed that acute stress induction by OFT was successful. A shift towards ambilaterality was detected in dogs after acute stress. Results also showed a significantly lower absolute laterality index in the chronically stressed dogs. Moreover, the direction of the first paw used in FRT was a good predictor of the general paw preference of an animal. Overall, these results provide evidence that both acute and chronic stress exposure can change behavioral asymmetries in dogs.

## Introduction

Over the last decades, a substantial increase in research on functional cerebral asymmetries in animals has taken place^1–3^. Domestic dogs (*Canis lupus familiaris*) are one of the key species which have been studied to elucidate the evolutionary and biological mechanisms of lateralization and, thus, to better understand the functional significance of lateralization for vertebrate animals in general^4,5^. Their long common evolutionary history with humans has contributed to complex socio-cognitive skills, similar behavioral characteristics, and abilities to humans^6,7^. Moreover, certain anatomical features specifically for dog–human communication have been developed^8,9^. Dogs are therefore considered excellent models for understanding the effects of domestication on the animal brain^10^.

Domestic dogs display different forms of behavioral asymmetries such as asymmetric tail wagging^11^, nostril preference^12^, visual^13,14^ and auditory processing^15^, as well as paw preferences^3,16^. Pawedness is one of the most known expressions of behavioral lateralization in dogs, similar to handedness in humans. A recent meta-analysis on dogs concluded that 68% of the dogs had either right or left paw dominance and, thus, showed individual-level asymmetry^3^. Measuring pawedness in dogs is a subject of interest not only to understand the evolutionary mechanisms of behavioral asymmetries but also to evaluate its potential use as a temperament and stress indicator in dogs^16,17^. Recent studies show that the direction of lateralization can be important to assess the stress reactivity of an individual dog. For example, some authors report a relationship between left-pawedness and greater reactivity to stress response, as well as a more pessimistic character in dogs^1,17^. Several different studies showed that measuring the strength of lateralization may also be relevant to understanding the behavioral and emotional characteristics of dogs^2,11,14,18^. For instance, dogs with stronger paw preferences exposed to novel stimuli and unfamiliar environments displayed more confident and relaxed behavior^18^. Weaker lateralization has been identified as a contributing factor to susceptibility to stress, anxiety, fear, and phobia in dogs^2,19^.

Brain asymmetries enable animals to process and integrate information effectively and accurately^20^. Thus, changes in cerebral asymmetries may manifest themselves with pathologies in emotional and mental processes as well as in behavior. Although some authors argue that cerebral asymmetries are primarily determined by genetic factors^21^, there is increasing evidence that they can also be altered in the short and/or long term by environmental factors, as cerebral asymmetries show plasticity to some extent^22^. Stress is, for instance, known to be related to functional and structural changes in cerebral asymmetries^23,24^. It has been suggested that stress regulation is mediated by the right hemisphere to a larger extent than by the left hemisphere^25^. Altered cerebral asymmetries due to stronger activation of the right hemisphere were reported in cases of stress in humans^23,26,27^. Acute stress-induced right hemispheric activation was also reported in domestic dogs in a study investigating head-orienting responses to different types of acoustic stimuli^15^.

Acute stress is mostly adaptive and protective as it initiates neuroendocrine, autonomic, and species-specific defense responses to help the individual cope with the stressor^28^. Increased activation of the sympathetic adrenomedullary (SA) axis manifests itself with autonomic responses such as increased heart and respiratory rates, whereas increased secretion of cortisol is a significant marker of the hypothalamic–pituitary–adrenal (HPA) activation^29,30^. Cortisol secretion is particularly important for acute stress response since it helps to mobilize the metabolic energy needed to cope with short-term stressors in the short term^31^. Cortisol also helps to normalize hypothalamic–pituitary–adrenal function and return it to a pre-stress state via a receptor-mediated negative feedback mechanism after the stressor is removed. Prolonged cortisol elevations caused by chronic stress, on the other hand, contribute to maladaptive changes in the body and the development of various disease states^32^. One manifestation of the adverse effects of continuous cortisol exposure is stress-induced structural changes in the corpus callosum which is one of the key structures in maintaining laterality^33,34^. Stress-induced reduction in the volume of the corpus callosum was suggested as the main cause of weakened cerebral asymmetries in individuals exposed to chronic stress^23,35^. It is well known that chronic stress plays an important role in the onset of various psychiatric disorders^36^. For several decades, atypical cerebral asymmetries have been reported in patients with psychiatric conditions^37–42^. It has been suggested that long common history with humans has not only positive but also some negative effects on dogs. Accordingly, humans and dogs share the same psychiatric disorders such as anxiety disorders, post-traumatic stress disorder, and obsessive–compulsive disorder with similar underlying pathological mechanisms^43^. Chronic exposure to stress is considered one of the most common reasons for developing such emotional disorders in dogs similar to humans^44,45^. Thus, studying the effect of stress on cerebral asymmetries may give significant insights into the functional mechanisms sustaining lateralization and possible stress-related changes in behavioral asymmetries in dogs. A recent research study on environmental conditions in dogs predicted that chronic stress may cause altered asymmetries in dogs similar to humans^16^. A recent study by Garbiec et al.^46^ demonstrated that dogs with left paw preference are more susceptible to acute stress. No comprehensive study has yet, however, investigated the possible effects of acute and chronic stress on behavioral asymmetries separately by comparing motor laterality of the same group of dogs before and after stress exposure. Considering this gap, this study intends to assess the short- and long-term effects of stress on behavioral asymmetries in dogs. Accordingly, behavioral asymmetries were aimed to assess in dogs after acute stress exposure and also in dogs with conditions that cause them to experience chronic stress. Suffering from emotional and behavioral disorders for a long period of time^47^, having a chronic pain condition^45^ and displaying certain behaviors in a repetitive fashion or depression-related behaviors^48^ were considered as indicators of chronic stress in dogs.

In this study, in order to create an acute stress environment, the Open Field Test (OFT) was used as a stressor arising from a socially isolated novel environment for the dogs^49,50^. Movement and postural asymmetries as well as the activity levels of the dogs were further aimed to measure during the OFT by using the DeepLabCut. DeepLabCut is a machine-learning software, which was used for postural estimation in humans and non-human animals^51,52^. It was used for movement analyses in different animal species such as primates^52^ and equines^53^. Deep-learning-based approaches have several advantages such as objective behavioral tracking, facilitated quantitative behavioral analyses, and, thus, reduced human labor during analyses^51,54,55^. Considering the advantages and recent applications of DeepLabCut, it was determined to use this method for measuring behavioral asymmetries and activity levels displayed by the dogs during the OFT.

This is the first study to investigate the effect of acute and chronic stress on the direction and strength of motor laterality in a large sample group of dogs by using two different laterality tests: the Kong Test (KT) and the Food-Reaching Test (FRT). Although KT is the most widely used method for determining paw in dogs^16,18,56–58^, some authors state that using this test to measure the direction of laterality may be misleading^59^. Behavioral laterality is mostly assessed by using food-reaching tasks in different animal species^60–64^. It was hypothesized that the FRT is a more reliable and applicable test to measure paw preference in dogs in comparison to KT. With this study, the main indicators of the hypothalamic–pituitary–adrenal and SA axis such as cortisol, heart rate, and respiratory rate were measured concomitant to motor laterality tests for the first time in the literature. The main hypothesis of this study was that both acute and chronic stress caused a shift toward ambilaterality in dogs. Moreover, it was hypothesized that measuring behavioral laterality can give significant information about chronic stress exposure and, thus, poor welfare conditions of dogs.

## Results

### Physiological measures

#### Cortisol levels

**Figure 1:**
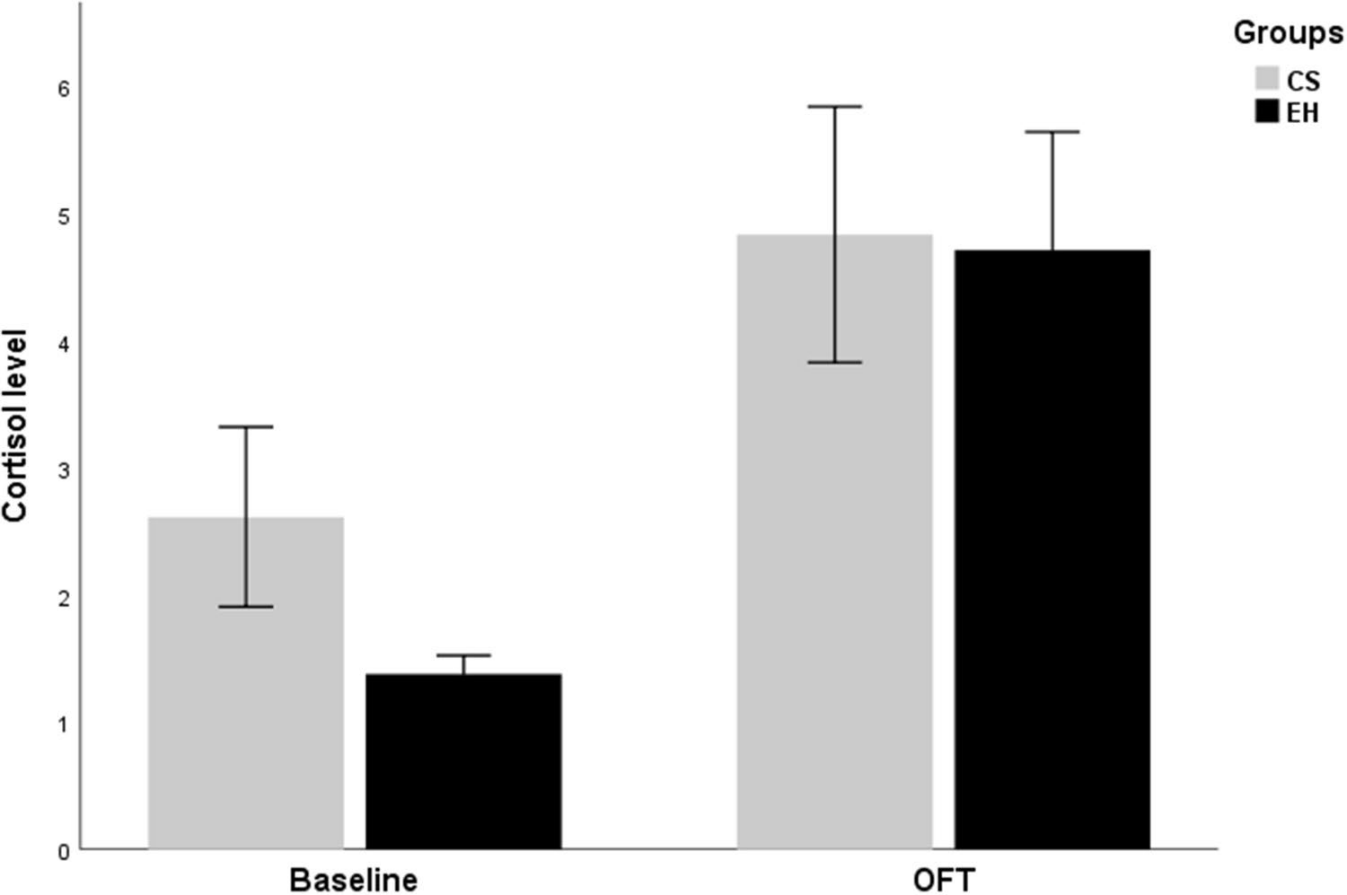
Cortisol levels in ng/mL for chronically stressed dogs (CS) and emotionally healthy dogs (EH) for the baseline and Open Field Test (OFT) conditions. Mean values are depicted, and error bars indicate standard errors.

Cortisol data were available for 18 chronically stressed (CS) and 22 emotionally healthy (EH) dogs (Fig. 1). The results of the ANOVA revealed a main effect of condition (F_(1,38)_ = 13.73; p < 0.01; χ^2^ = 0.27), indicating higher cortisol levels after the OFT (4.78 ng/mL ± 0.69) than after baseline (2.00 ng/mL ± 0.33). The main effect of the group (p = 0.39) and the interaction group × condition (p = 0.46) failed to reach significance.

#### Heart rates

Heart rate data were available for 21 chronically stressed (CS) and 27 emotionally healthy (EH) dogs. Statistical analysis revealed a main effect of the condition (F_(2,45)_ = 18.92; p < 0.001; χ^2^ = 0.29), indicating higher heart rates after the OFT (123.63 HR/min ± 3.00) than after baseline (102.68 HR/min ± 3.11) or before the OFT (108.23 HR/min ± 3.15). This indicates that the stress induction was successful. The main effect of the group (p = 0.48) and the interaction group × condition (p = 0.48) failed to reach significance.

#### Respiration rates

Respiratory rate data were available for 19 chronically stressed (CS) and 27 emotionally healthy (EH) dogs*.* The results of the ANOVA revealed a main effect of the condition (F_(2,43)_ = 15.66; p < 0.001; χ^2^ = 0.26), indicating higher respiration rates after the OFT (93.97 ± 7.10) than after baseline (53.79 ± 4.05) or before the OFT (69.79 ± 7.49). This indicates that the stress induction was successful. The main effect of the group (p = 0.77) and the interaction group × condition (p = 0.42) failed to reach significance.

### Paw preferences in the baseline condition

#### Food-reaching test

Most of the dogs (47/60; 78.3%) completed the FRT in the non-stressful baseline condition at home. According to the results of this test, 27.7% of the dogs were right-pawed, 31.9% were left-pawed, and 40.4% were ambilateral. The mean LI (Laterality Index) was 0.05 (± 0.08). A one-sample t-test indicated that this LI was not significantly different from zero (t_(46)_ = 0.67; p = 0.51), indicating that there was no population-level asymmetry towards one side in this test.

#### Kong test

Overall, 38 dogs (63.3%) completed the KT in the non-stressful baseline condition at home, less than for the FRT. According to the results of this test, 28.9% of the dogs were right-pawed, 23.7% left-pawed, and 47.4% ambilateral. The mean LI was 0.0052 (± 0.07). A one-sample t-test indicated that this LI was not significantly different from zero (t_(37)_ = 0.07; p = 0.94), indicating that there was no population-level asymmetry towards one side in this test.

#### Sex differences in paw preferences

The comparison failed to reach significance for both the FRT (p = 0.99) and the KT (p = 0.72). This indicates that there were no statistically significant sex differences in paw preferences in the present sample.

#### Effect of sterilization status on paw preferences

The sterilization status of the only 2 dogs was unknown. As sex differences in paw preferences in dogs may be influenced by sterilization status, we used independent-sample t-tests to compare the LIs of sterilized and non-sterilized dogs for both tests. The comparison failed to reach significance for both the FRT (p = 0.94) and the KT (p = 0.95). This indicates that there was no statistically significant effect of sterilization status on paw preferences in the present sample.

### Effects of stress on paw preferences

#### Effects of stress on the LI

LIs were evaluated using 2 × 2 mixed ANCOVAs with the between-subjects factor group (CH dogs/EH dogs) and the within-subjects factor condition (baseline/OFT). Sex was included as a covariate. All effects failed to reach significance for both the FRT (all p’s > 0.57) and the KT (all p’s > 0.41).

#### Effect of stress on the strength of lateralization

The results of the ANCOVA revealed a significant main effect of group (F_(1,40)_ = 4.50; p < 0.05 χ^2^ = 0.10), indicating a significantly lower absolute LI (0.35 ± 0.06) in the CS dogs than in the EH dogs (0.53 ± 0.06). All other effects failed to reach significance (all p’s > 0.40).

Absolute LIs in the KT were also evaluated using a 2 × 2 mixed ANCOVA with the between-subjects factor group (CS dogs/EH dogs) and the within-subjects factor condition (baseline/OFT). Sex was included as a covariate. All effects failed to reach significance (all p’s > 0.44).

#### Effect of stress on the direction of paw preferences

##### FRT

Table 1 shows how the distribution of paw preferences in the FRT and the KT changed after the dogs performed the OFT. Results indicate a shift towards ambilaterality after the OFT. While the proportions of the three preference types (ambilateral, left, and right) did not differ from an equal distribution in the baseline condition for both tests (binomial test against an equal proportion of 0.33333), the ambilateral condition differed significantly from an equal distribution after the OFT. For the FRT, about 40% of dogs were ambilateral in the baseline condition, but 48% were ambilateral in the OFT condition. For the KT, 47% of dogs were ambilateral in the baseline conditions and about 59% were ambilateral in the OFT condition. The datasets including group, LI, Z-score per animal and individual paw preferences were also shown in Supplementary Table 1 for two tests in both conditions.

**Table 1:** The distribution of paw preferences in the Food-Reaching Test (FRT) and the Kong Test (KT) for the baseline and the stressful OFT condition.

Condition	Preference	Number	Total n	Proportion (%)	p-value
FRT Baseline	Ambilateral	19	47	40	0.35
	Left	15	47	32	1.00
	Right	13	47	28	0.44
KT Baseline	Ambilateral	18	38	47	0.08
	Left	9	38	24	0.23
	Right	11	38	29	0.61
FRT OFT	Ambilateral	25	52	48	0.03*
	Left	15	52	29	0.55
	Right	12	52	23	0.14
KT OFT	Ambilateral	20	34	59	0.003**
	Left	8	34	23	0.28
	Right	6	34	18	0.07

P-values indicated the p-value of a binominal test against equal distribution (proportion = 0.33333). Asterisks indicate p-values with *p < 0.05, **p < 0.01.

### Correlations between FRT and KT

Table 2 shows the correlations between the LI in the FRT and the LI in the KT in the non-stressful baseline condition at home and after the OFT. The correlation between the FRT LI and the KT LI failed to reach significance for the baseline condition (r = − 0.07; p = 0.70), as well as for the OFT condition (r = − 0.15, p = 0.42). Interestingly, there was a significant positive correlation between baseline and OFT condition for the FRT (r = 0.69; p < 0.001). This indicated that dogs with a more rightward or leftward preference in one condition also showed a similar preference in other conditions. Such an effect was not found for the KT.

**Table 2:** Correlations between the FRT and KT for the LI.

	FRT baseline	FRT OFT	KT baseline	KT OFT
FRT baseline	1			
FRT OFT	0.69***	1		
KT baseline	− 0.07	0.12	1	
KT OFT	0.13	− 0.15	0.12	1

Numbers indicate correlation coefficients.

Asterisks indicate p-values with ***p < 0.001.

For the absolute LI (Table 3), the non-stressful home condition showed a significant correction with the stressful OFT condition for the FRT (r = 0.49; p < 0.001) and the KT (r = 0.41; p < 0.05). All other correlation coefficients failed to reach significance (all p’s > 0.16).

**Table 3:** Correlations between the FRT and KT for the absolute LI.

	FR baseline	FR OFT	KT baseline	KT OFT
FR baseline	1			
FR OFT	0.49***	1		
KT baseline	0.06	0.25	1	
KT OFT	0.02	0.4	0.41*	1

Numbers indicate correlation coefficients.

Asterisks indicate p-values with *p < 0.05, ***p < 0.001.

### Relation between the first paw use and LI

Since many studies on dog laterality often only use one or very few trials, we added another analysis to investigate the predictive power of the first trial on the overall LI. To this end, we used between-subjects t-tests with the direction of the first paw use (left or right) as a group factor. For FRT, the comparison reached significance for both the baseline condition (t_(42)_ = − 3.91; p < 0.001; Cohen’s d = − 1.21) and the acute stress condition (t_(49)_ = − 3.22; p < 0.01; Cohen’s d = − 0.90). Similarly, for the KT, the comparison reached significance for both the baseline condition (t_(42)_ = − 3.91; p = 0.043) and the acute stress condition (t_(28)_ = − 2.12; p < 0.05; Cohen’s d = − 0.82).

### Behavioral analysis of the OFT data using DeepLabCut

**Figure 2:**
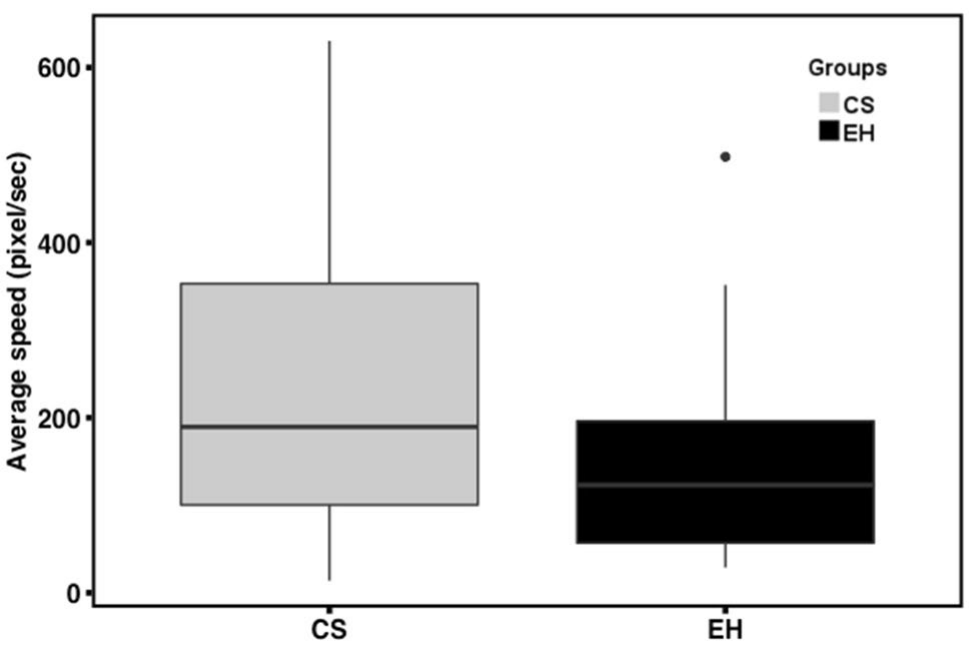
Comparison of average movement speed between groups. The solid line indicates the median. The bottom of the box represents the first quartile (25th percentile) and the top of the box represents the third quartile (75th percentile). Whiskers represent the lower and upper bound for data excluding outliers.

**Figure 3:**
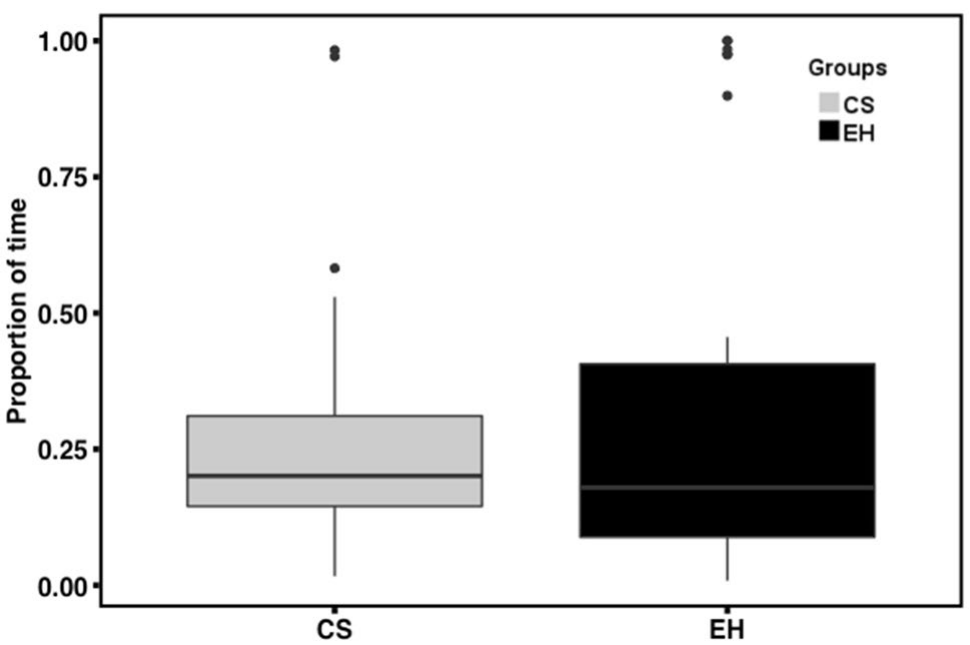
The proportion of time spent nearby novel object between groups. The solid line indicates the median. The bottom of the box represents the first quartile (25th percentile) and the top of the box represents the third quartile (75th percentile). Whiskers represent the lower and upper bound for data excluding outliers.

The average movement speed in the OFT showed a significant difference between the two groups of dogs (t_(49.09)_ = 2.29, p = 0.03, Cohen’s d = 0.59), indicating higher general activity levels in CS dogs, as compared to EH dogs (Fig. 2). In contrast, the proportion of time spent nearby a novel object was not significantly different between groups (t_(47.78)_ = − 0.81, p = 0.42, Cohen’s d = 0.22) (Fig. 3). Moreover, there was no significant difference in resting between groups (t_(49.12)_ = − 1.46, p = 0.15, Cohen’s d = 0.36). However, a significant difference in the amount of running was detected (t_(44.44)_ = 2.12, p = 0.039, Cohen’s d = 0.60), indicating a greater amount of running in the CS dogs in the OFT.

**Figure 4:**
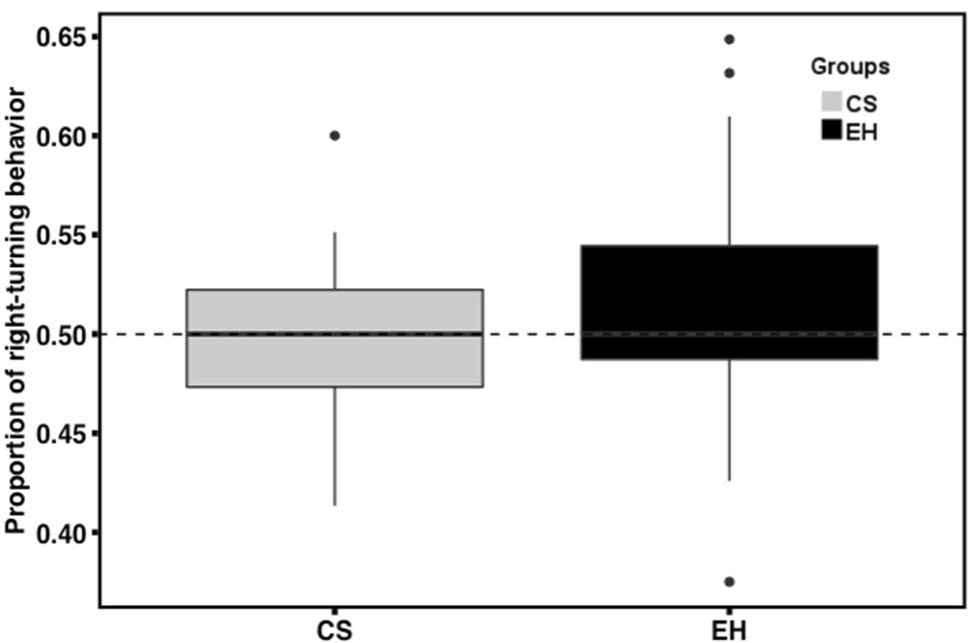
Comparison of proportion of turning bias between groups. The solid line indicates the median. The bottom of the box represents the first quartile (25th percentile) and the top of the box represents the third quartile (75th percentile). Whiskers represent the lower and upper bound for data excluding outliers.

There was no significant behavioral asymmetry in turning behavior (t_(53.28)_ = 1.29, p = 0.20, Cohen’s d = 0.33) (Fig. 4). To examine the effect of the stress on the asymmetry of curiosity-driven behavior, the proportion of left–right turning during approaching a novel object was compared between groups. No significant difference was observed (t_(50.14)_ = 1.50, p = 0.13, Cohen’s d = 0.40).

Correlational analysis was performed between movement speed and both baseline and acute cortisol levels. In both CS and EH dogs, no significant correlations were found for baseline cortisol levels (CS dogs: r = − 0.03; p = 0.91; EH dogs: r = − 0.24; p = 0.25). In contrast, a significant negative correlation between cortisol levels after acute stress and average movement speed was found in EH dogs (r = − 0.52; p = 0.01). This effect was not observed in CS dogs (r = − 0.12; p = 0.61). This suggests that in EH dogs, the less stressed individuals showed more active responses in the Open Field Test. However, no such effect was observed in CS dogs.

To further explore this effect, correlational analyses were performed separated by sex, age, and sterilization status. For sex, a significant correlation between cortisol levels after acute stress and movement speed was observed in male dogs (r = 0.58; p = 0.01), but not in female dogs (p = 0.12). For baseline cortisol levels, both correlations failed to reach significance (all ps > 0.25). For age, none of the correlations with cortisol levels reached significance in both group (all ps > 0.3), suggesting that age does not affect cortisol levels. For sterilization status, a significant correlation between cortisol levels after acute stress and movements speed was observed for both non-castrated animals (r = 0.57; p = 0.01) and castrated animals (r = − 0.51; p = 0.02). For non-castrated animals, higher cortisol levels were related to higher movement speeds, while for castrated animals, higher cortisol levels were related to lower movement speeds. For baseline cortisol levels, both correlations failed to reach significance (all ps > 0.36).

## Discussion

This study is the first to examine the relationship between stress and motor laterality in a large sample group of dogs considering physiological and behavioral parameters. Although salivary cortisol was found to be significantly higher after the OFT, one should consider that cortisol has some disadvantages in dogs as a stress marker since circadian rhythm, individual variability and external stressors may affect the results^65,66^. Therefore, the measurement of supplemental stress parameters such as heart and respiration rate is advised in dogs^61^. In our study, the heart and respiratory rates of the dogs were also significantly higher after the OFT. Taken together, the results for these parameters are clearest that the OFT successfully induced stress in the dogs as expected^67^.

One of the main objectives of this study was to investigate the effect of stress on functional cerebral asymmetry (FCA) in dogs. Increased level of ambilaterality, i.e., a shift in the direction of lateralization was observed in the dogs in case of acute stress caused by the OFT situation. Dogs have individual-level asymmetry and, thus, ambilaterality is not common in dogs, as shown in different studies including this study^63,64^. This result might be explained by the increased activity of the right hemisphere, as right hemispheric activity was shown in different animal species including dogs in case of unexpected and novel stimuli^15,23,68^. The other key finding of this study was the demonstration of reduced FCA in CS dogs in the FRT. Chronic stress-induced atypical behavioral asymmetries were previously reported in rats^69^ and also in a small group of dogs^16^. However, it is the first manifestation of altered behavioral asymmetries in CS dogs in a systematic study using a large sample size. Mundorf et al.^69^ suggested that stress level is an important factor to cause alterations in behavioral asymmetries as atypical behavioral asymmetries were only detected in cases of high chronic stress in rats. The relationship between a high level of chronic stress and psychiatric diseases as well as between ambilaterality and various psychiatric conditions such as phobias and PTSD were manifested in humans^70,71^. Considering these studies, it is not surprising that dogs exposed to a certain level of chronic stress show weakened FCAs. This finding is promising in terms of potential application to clinical veterinary science as dogs and humans share similar brain pathologies of psychiatric diseases^72^. So far, various studies claim that dogs with weaker motor lateralization are more sensitive to stress and, thus, are more fearful and excitable^2,18^. Considering the findings of the study, however, one may suggest that weak laterality is not the cause but the result of chronic stress in dogs similar to humans^73^.

The results of this study showed no significant correlation between KT and the FRT regarding either the strength or direction of paw preference. Although KT is the most common method to measure paw preference in dogs, there are discussions that this test may not be reliable since dogs tend to use their paws randomly to stabilize the Kong toy^74^. It can be assumed that the FRT, which includes a fixed (non-moving) test device and requires skilled reaching to get the food, gives more reliable results than the KT. Moreover, although the result was not significant, it was observed that the number of dogs interested in the FRT was higher than the number of dogs interested in the KT. This may also support our hypothesis that the FRT can be a more ideal test to measure paw preference in dogs.

This study supports previous reports of skilled paw use in dogs^75,76^. Even though dogs do not use their paws as skillfully as humans, having a dominant paw/limb may bring many benefits to them. For instance, gray wolves, which are the closest relatives of dogs, use their paws for hunting small prey such as rabbits and squirrels. Thus, since hunting was one of the pioneer reasons for the selective breeding of dogs, the trait for skillful paw use might have been passed on from one generation to another from ancestor wolf to domestic dog. This is particularly true for small breeds which are still used for burrow hunting^77^. Considering those evolutionary mechanisms behind pawedness in dogs, it can be argued that dogs are more easily adapted and motivated to FRT. In addition to LIs, absolute LIs were calculated as an indicator of the strength of lateralization, regardless of preference for direction in this study. Since dogs have a higher rate of left-pawed animals than left-handed individuals do occur in humans and since left-pawed and right-pawed animals may cancel each other out (e.g., if a reduction of asymmetry occurs in one condition) for the LI effects, the absolute LI may be more informative in this species than the LI. In line with the meta-analysis study^3^, dogs showed no population level of asymmetry in both tests.

Different studies reported a significant sex effect on the direction of paw preferences in dogs^1,75,78^. Results of this study, however, revealed no significant effect of sex on FCA in dogs which is in line with the recent meta-analysis^3^. Moreover, no significant effect of sterilization status on FCA was determined in the study dogs. The contradicted results between the studies may be attributed to different methodological approaches. For instance, in the study by Wells^78^ in which different tests were used, the distribution of paw preferences differed between the tests. In a previous study, on the other hand, using a “Test Your Pet” Method by unprofessional people might increase the risk of having unreliable data^75^. The method used in this study seemed to overcome the limitations of the previous studies as the standard FRT was used as suggested by the previous meta-analysis^3^.

To the best of our knowledge, the association between first and overall paw usage in dogs has been evaluated for the first time in this study. The results show that first paw use and overall paw use are significantly associated. Thus, first paw use can be a good indicator of the overall paw preference of the dogs. Moreover, this finding is consistent with the studies indicating that the first hand/paw preference in the first trial is consistent with general hand preferences in macaques^79^ and cats^63^. Although the first paw preference may be an indicator of general paw preference, the lack of correlation between FRT and the KT in terms of direction and strength of paw use indicates that paw preference in dogs may be task-dependent. This result supports the results of a study using different motor laterality tests to investigate the stability of motor bias in dogs^80^. Thus, further studies exploring this correlation and task dependency of laterality in dogs may have particular importance in adding information to this field.

In this study, DLC was used for the first time while analyzing the behaviors of dogs during the OFT. The directional bias especially while approaching a foreign object was evaluated with DLC for the first time in this study. The only significant result of the DLC analyses was related to the activity. The dogs in the CS group were more active during the OFT than the healthy group in general. Although this seems to indicate that this group is more prone to show an active/proactive strategy^81^, it should be taken into account that the majority of dogs in the CS group are working dogs. Moreover, less stressed individuals in the group of EH dogs showed more active responses in the OFT. Although these results seem to contradict each other, they may actually be related to the different activity types of dogs. CS dogs may have a higher tendency to exhibit stereotypical, i.e., abnormal repetitive behaviors or depressive behaviors, i.e., loss of interest in exploration and environment while the less stressed dogs in the EH group can be the ones who display more exploratory behaviors. However, further analyzes are required to reach a definite decision. DLC analyses also revealed that males and non-castrated dogs display higher activity levels in the OFT. These results are rather expected as male dogs are known to be more active than females^82^ and sterilization may reduce the general activity level of the dogs^83^.

In conclusion, this study is the first study investigating changes in behavioral laterality in dogs with respect to acute and chronic stress. This study confirmed that altered FCAs can be considered indicators of poor welfare in dogs as proposed in our previous study^16^. Further studies investigating the relationship between psychiatric disorders and FCA would be beneficial to understand the underlying mechanisms and clinical manifestations of these disorders in domestic dogs.

## Materials and methods

### Animals and location

A total of 60 dogs (24 males, 36 females) between 8 months and 9 years (average age: 36 months) were tested in the present study. The announcement of the study was made through veterinary clinics, the internet, and social media (e.g.; Instagram), targeting the dog owners in the Ankara region. Owners of pet dogs (n = 42) voluntarily attended this study. Working dogs (n = 18) were recruited from the Turkish Ministry of Commerce, Dog Training Center. Two groups of dogs, i.e., chronically stressed (CS) (n = 28) and emotionally/physically healthy (EH) dogs (n = 32) were determined by a veterinary behavior specialist based on individual interviews with dog owners, and behavioral assessments during the first introduction. At least one of the following criteria had to be met along with the evaluation of behavioral history to confirm the chronic stress in dogs: (i) Long-term emotional/behavioral disorders related problems, (ii) Chronic disease state and/or chronic pain, (iii) Manifestation of abnormal repetitive behaviors. Chronic disease state and/or chronic pain, (iii) Manifestation of abnormal repetitive behaviors. Increased level of displacement behaviors was reported in challenged chronically stressed dogs^84^. The dogs that consistently showed displacement behaviors during the behavioral observation, such as body shaking, paw lifting, and yawning in addition to a lowered body posture were also included in the CS group as those behaviors were indicators of negative emotional state and inability to recover from a challenged situation.

The model for chronic stress was identified as 6 weeks of social and spatial restriction in dogs^48^. All working dogs (n = 18) tested in this study were kept in individual cages for a long period of time (minimum of 6 months) with limited access to social interaction and mental activities (only during training sessions). They did not receive any mental stimulation, toys, or comfortable places to rest in the cage environment, nor did they engage in any regular or scheduled social interactions with their owners. Based on the welfare assessment, behavioral observations and the relevant literature^48,85^, working dogs were included in CS group in this study. All procedures have been conducted in accordance with the ARRIVE guidelines and Ethical approval was obtained from the Animal Experiments Local Ethics Committee, Ankara University (2018-4-39). Informed consent was obtained from the dogs’ owners prior to the study. All methods were performed in accordance with the relevant guidelines and regulations.

### Experimental procedure

Except for the OFT, the same procedure was conducted in two different environments, i.e., a stress-free home environment and a novel environment for each dog. The stress-free home environment was the home environment for the pet dogs while the cage environment was for the working dogs. During all home tests, testing persons wait until dogs become habituated to the new person in their environment. The habituation period was a maximum of 30 min. Dogs with a habituation period exceeding 30 min were excluded from the study. A preliminary assessment for each dog was made according to the statements of the owners before the testing session. None of the anxious/fearful dogs or with aggression problems were included in the study. The main criteria for habituation were defined as follows: (i) Wait until the activity of the dog and greeting behavior is normalized and (ii) Wait until panting stops and the normal respiratory pattern is observed.

#### Acute stress context

OFT was carried out as a way to induce mild acute stress in dogs. It was conducted at the Behavioral Unit of Ankara University Faculty of Veterinary Medicine. The dogs were left alone in a room with a rectangular floor area of 14 m^2^ and were recorded with four GoPro Hero 7 cameras (1920 × 1440-pixel resolution) for 5 min^86^. They were monitored from a separate observation room where the dogs could not see the other side. A remote-controlled toy car with sound and motion features was used as the novel object during the last 1 min of the test. Any physical contact between the novel object and the dogs was avoided.

#### Physiological data collection

The heart and respiratory rate of each animal were measured using a clinical stethoscope in the home environment before the behavioral tests and considered basal values. The same procedure was repeated in the novel environment immediately after the OFT and used as acute stress parameters. Additionally, saliva samples of dogs were collected by using a Sarstedt^®^ Salivette cotton swab in both environments to measure the cortisol levels of the dogs. All saliva samples were collected at the same time interval, i.e., between 10 and 12 a.m. Like other physiological measures, the saliva sample taken in the home environment was used as basal cortisol value, while the one collected 10 min after the OFT was used as an indicator for acute stress. It has previously been shown that approximately 10 min is needed after the induction of a stressor for a detectable increase in peripheral cortisol to occur^76^. Saliva samples were transported at + 4 °C in sample containers and stored at – 20 °C until cortisol analysis. In order to avoid any food residue in the mouths of the dogs, dog owners were asked to restrict the food intake of their dogs starting two hours before cortisol sampling.

### Motor laterality tests

**Figure 5:**
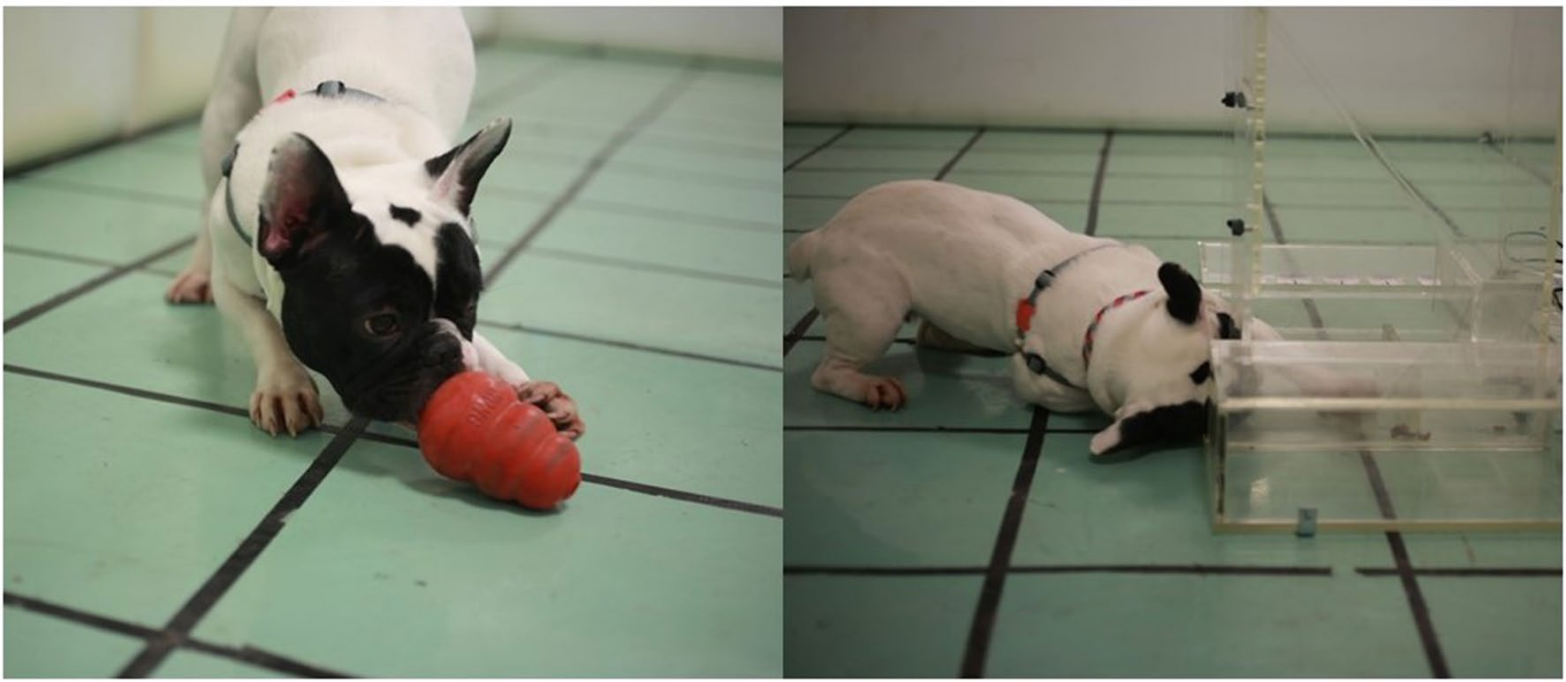
A dog performing Kong Test (on the left side) and Food-Reaching Test (on the right side).

After the physiological data collection was completed, a FRT and KT were used as motor laterality tests (Fig. 5). Both motor laterality tests were terminated when the dogs reached 50 paw responses which were counted by the experimenter in real-time. The behavior of the dogs was video-recorded continuously during test sessions using GoPro Hero 7 cameras placed at different angles. Half of the dogs were tested in the home environment first, whereas the other half was tested in the novel environment first to eliminate the possible environmental effects on laterality results. FRT was introduced as the first motor laterality test to dogs in both environments. KT was performed immediately after the FRT. Dogs did not receive any form of positive reinforcement such as social (e.g., verbal praise) and/or tactile (e.g., petting) interactions during the experimental procedure.

#### Food-reaching test

The Food-Reaching testing apparatus used in the current study is a stable, transparent, and adjustable device, allowing all dog breeds regardless of their body and paw sizes. The dog could see the treat but was able to reach it only by using its paws. The experimenter positioned herself/himself behind the testing device and offered the food reward from the top of the device which later moved to the bottom part. To motivate the animals to experiment, a small piece of food reward was offered to dogs by the experimenter as an introductory step at the beginning of each session. FRT was performed to determine the paw preferences of the dogs.

#### Kong test

The commercially available small or large Kong toy was used according to the subject's size to determine the paw preferences of the dogs. Within the scope of this study, wet food was filled into the Kong toy and placed in the freezer at least 3–4 h before the test. The Kong toy was presented to the dog on a flat surface in the environment to be tested.

### Data analysis

#### Analysis of physiological parameters

Cortisol levels (see Fig. 1) were evaluated using a 2 × 2 mixed ANOVA with the between-subjects factor group (CS dogs/EH dogs) and the within-subjects factor condition (baseline/OFT). Heart and respiratory rates were evaluated using a 2 × 3 mixed ANOVA with the between-subjects factor group (CS dogs/EH dogs) and the within-subjects factor condition (baseline/before OFT/after OFT). Cortisol levels were assayed using a commercially available competitive ELISA kit (Brand, Model, cat no). HUMAN brand COMBI WASH model washing device and NEXT LEVEL brand ALİSEİ model reading device were used. Cortisol was measured in ng/mL.

#### Behavioral analysis for motor laterality tests

The video footage of the dogs obtained from the motor laterality tests was analyzed by two trained and blinded raters. The percentage of interobserver reliability was always higher than 90%. To quantify individual paw preferences, an LI (laterality index) was determined using the formula LI = (R − L)/(R + L). R indicated the number of right paw uses and L the number of left paw uses. The LI had a range from − 1.0 (left paw use only) to + 1.0 (right paw use only). In addition to LIs, absolute LIs (absolute values of each Laterality Index) were calculated as an indicator of the strength of lateralization, regardless of preference for direction. Since dogs have a higher rate of left-pawed animals than left-handed individuals do occur in humans, the absolute LI may be more informative in this species than the LI, since the LI effects for left-pawed and right-pawed animals may cancel each other out (e.g., if a reduction of asymmetry occurs in once condition).

Whether or not dogs showed a significant bias in one direction for paw preference on the individual level was determined using binomial Z-scores for each dog [z = (R − 0.5 N)/√(0.25 N)]. According to the equation, N refers to the total number of paw scores, while R indicates the number of right paw scores. Dogs with a positive Z-score value (z ≥ 1.96) were scored as R-pawed, whereas those with a negative Z-score value (z ≤  − 1.96) were scored as L-pawed. Dogs with Z-scores between + 1.96 and − 1.96 were classified as ambilateral.

Absolute LIs were calculated to investigate the strength of lateralization under stress, independent of the direction of lateralization. Absolute LIs in FRT were evaluated using a 2 × 2 mixed ANCOVA with the between-subjects factor group (CS dogs/EH dogs) and the within-subjects factor condition (baseline/OFT). Sex was included as a covariate. Independent-samples t-tests were used to compare the LIs of male and female dogs for both tests. The alpha value for significance was set at 0.05. IBM SPSS Statistics 27 was used for all statistical analyses.

#### Behavioral analysis of the OFT by DeepLabCut

In order to get a deeper insight into dog behaviors in the OFT were evaluated through the movement trajectories captured with ‘DeepLabCut’, an open-source python toolbox using deep neural network^51^. Frame-by-frame body positions were tracked with DeepLabCut (Fig. 5). The extracted x–y coordinates were smoothed using the spline function to remove the effect of tracking failure before analysis. Two metrics were calculated from the stream of body movement: average movement speed and distance to the novel object. The movement speed was considered as a proxy of an activity level in the Open Field Test, similarly to the previous study^82^. It was defined and computed as moment-to-moment subtraction between successive trajectories of each video frame. Next, the average distance from the body to the novel object was quantified, which reflects the approach and avoidance tendency during the OFT. That is if the novel object invokes curiosity, or conversely, fear, animals might show approach or avoidance responses to it. These behaviors must be reflected in the distance metric to the object. Thus, body-to-object distance from obtained x–y coordinates was calculated (Supplementary Video 1).

Furthermore, animals typically show distinct patterns between active and pause states in the speed profile over the time course of the OFT. Therefore, active, and resting patterns were classified, and for each dog, the proportion of time in each behavioral state was determined. For this purpose, a three-state Hidden Markov model was applied to the continuous trajectory of the speed profile. Briefly, the Hidden Markov Model is an unsupervised time-series model to classify input data into discrete categories^87^ and has been demonstrated as a powerful method for the classification of animal behaviors^88,89^. In the present project, speed is classified into three states: running, marginal, and resting. Using these categories, the spending proportion of time resting and running states between groups were compared with each other.

The turning behavior of the dogs was also assessed using DeepLabCut. Turning behavior was defined from tracked trajectories, using the same method in Mundorf et al.^69^. Angular movements were measured as the angular difference of body-head orientation between two consecutive frames. The turning behavior was defined as cumulative angular movements of more than 45°. To prevent the double count (or, potentially more duplicated counts) of turning more than 90°, an occurrence of a turning behavior was defined, once the angular difference was smaller than 2°. The rationale of this criterion is that a small angular difference between frames indicates animals stopped their turning movement or started to move forward. Both left and right turning behaviors were separately counted and used for subsequent analysis.

### Statistical analyses

The parametric test was applied to normally distributed data from the behavioral analysis for motor laterality tests, while the non-parametric test was applied to data from the analysis of physiological parameters which deviated from normality. The data obtained from Behavioral Analysis of the OFT by DeepLabCut were tested for normal distribution using The Shapiro–Wilk test. The data of the average movement speed and the proportion of time spent in the OFT departure from normality and data were log-transformed. According to log transformation, a parametric test was applied to the average movement speed, while a non-parametric test was applied to the proportion of time spent in OFT. The data of turning behavior in OFT did not deviate from normality, and a parametric test was applied.

## Supplementary Information

Supplementary Table 1.

Supplementary Video 1.

## Data Availability

Since Turkish Government Working Dogs were tested in this study, data are available from the authors upon reasonable request and with the permission of the Republic of Turkey, Ministry of Trade.
