# Supplementary material for: Acute and chronic stress alter behavioral laterality in dogs
Source: Sci Rep. 2023 Mar 11;13:4092. doi: 10.1038/s41598-023-31213-7 (PMC10008577; doi:10.1038/s41598-023-31213-7)
Supplement: Supplementary file 1 — Supplementary Table 1. [file 41598_2023_31213_MOESM1_ESM.docx]

**Supplementary Data**

**Supplementary Video 1.** Dog tracking video during novel object approach in OFT

**Supplementary Table 1:** Individual paw preferences in dogs for two tests in both conditions [Group: (P: pet dogs, W: working dogs); LI: laterality index; Z-score: positive Z score values (Z ≥ 1.96) indicate right lateral bias; negative Z-score values (Z ≤ −1.96) indicate left lateral bias; Pref: paw preference (R: right pawed; L: left pawed; A: ambilateral); _: value not calculated due to absence of paw intervention

|  |  | *Baseline* | | | | | | *After OFT* | | | | | |
| --- | --- | --- | --- | --- | --- | --- | --- | --- | --- | --- | --- | --- | --- |
|  |  | **Food Reaching Test** | | | **Kong Test** | | | **Food Reaching Test** | | | **Kong Test** | | |
| Dog | **Groups** | **LI** | **Z-score** | **Pref** | **LI** | **Z-score** | **Pref** | **LI** | **Z-score** | **Pref** | **LI** | **Z-score** | **Pref** |
| 1 | P | -.56 | -3.96 | L | -.04 | -.28 | A | -.32 | -2.26 | L | .32 | 2.26 | R |
| 2 | P | .08 | .57 | A | -.24 | -1.70 | L | .16 | 1.13 | A | -.52 | -3.68 | L |
| 3 | P | -.16 | -1.13 | A | -.33 | -.58 | A | _ | _ | _ | _ | _ | _ |
| 4 | P | -.04 | -.28 | A | .32 | 2.26 | R | .48 | 3.39 | R | .00 | .00 | A |
| 5 | P | .00 | .00 | A | -.48 | -3.39 | L | -.60 | -4.24 | L | .32 | 2.26 | R |
| 6 | P | .20 | 1.41 | A | .20 | 1.41 | A | .24 | 1.70 | R | .00 | .00 | A |
| 7 | P | _ | _ | _ | .40 | 2.83 | R | 1.00 | 2.65 | R | _ | _ | _ |
| 8 | P | .17 | 1.18 | A | .28 | 1.98 | R | .00 | .00 | A | _ | _ | _ |
| 9 | P | _ | _ | _ | .96 | 6.79 | R | _ | _ | _ | .80 | 5.66 | R |
| 10 | P | .68 | 4.81 | R | .00 | .00 | A | .44 | 3.11 | R | _ | _ | _ |
| 11 | P | -.80 | -5.66 | L | -1.00 | -2.24 | A | -.88 | -6.22 | L | -1.00 | -1.00 | L |
| 12 | P | _ | _ | _ | _ | _ | _ | _ | _ | _ | .32 | 2.26 | L |
| 13 | P | -.50 | -1.73 | L | -.16 | -1.13 | A | _ | _ | _ | _ | _ | _ |
| 14 | P | .64 | 4.53 | R | _ | _ | _ | .20 | 1.41 | A | -.04 | -.28 | A |
| 15 | P | -.08 | -.57 | A | .52 | 2.40 | R | -.11 | -.75 | A | -.08 | -.39 | A |
| 16 | P | -.84 | -5.94 | L | -.04 | -.28 | A | -.92 | -6.51 | L | -.20 | -1.41 | A |
| 17 | P | 1.00 | 1.41 | A | -.17 | -.82 | A | 1.00 | 2.00 | R | .04 | .20 | A |
| 18 | P | .08 | .57 | A | .36 | 1.80 | A | .04 | .28 | A | .24 | 1.70 | A |
| 19 | P | .64 | 4.53 | R | -.45 | -1.51 | A | -.48 | -3.39 | L | -.28 | -1.98 | L |
| 20 | P | .68 | 4.81 | R | -.68 | -4.81 | L | .16 | 1.13 | A | 1.00 | 1.41 | A |
| 21 | P | -.48 | -3.39 | L | .69 | 2.50 | R | .20 | 1.00 | A | .14 | .38 | A |
| 22 | P | -.52 | -3.68 | L | _ | _ | _ | -.60 | -4.24 | L | -.25 | -.71 | A |
| 23 | P | _ | _ | _ | _ | _ | _ | _ | _ | _ | _ | _ | _ |
| 24 | P | .52 | 3.68 | R | .04 | .60 | A | _ | _ | _ | _ | _ | _ |
| 25 | P | -.72 | -5.09 | L | -.62 | -2.84 | L | -.56 | -2.89 | L | _ | _ | _ |
| 26 | P | .64 | 4.53 | R | .20 | 1.41 | A | 1.00 | 7.07 | R | -.36 | -2.55 | L |
| 27 | P | -.28 | -1.98 | L | _ | _ | _ | -.20 | -1.41 | A | .00 | .00 | A |
| 28 | P | _ | _ | _ | _ | _ | _ | _ | _ | _ | _ | _ | _ |
| 29 | P | _ | _ | _ | _ | _ | _ | -1.00 | -1.00 | A | _ | _ | _ |
| 30 | P | .17 | .93 | A | -.38 | -1.96 | L | -.50 | -1.00 | A | _ | _ | _ |
| 31 | P | -1.00 | -7.07 | L | -.40 | -2.83 | L | -.92 | -6.51 | L | .20 | 1.41 | A |
| 32 | P | -.84 | -5.94 | L | .44 | 3.11 | R | -.80 | -5.66 | L | .32 | 2.26 | R |
| 33 | P | .56 | 3.96 | R | _ | _ | _ | 1.00 | 2.00 | A | -1.00 | -1.41 | A |
| 34 | P | -.28 | -1.98 | L | _ | _ | _ | -.02 | -.13 | A | -.14 | -.93 | A |
| 35 | P | .17 | 1.13 | A | -.08 | -.57 | A | .32 | 2.26 | R | -.05 | -.32 | A |
| 36 | P | _ | _ | _ | _ | _ | _ | -.16 | -1.13 | A | 1.00 | 1.00 | A |
| 37 | P | _ | _ | _ | _ | _ | _ | -.96 | -6.79 | L | _ | _ | _ |
| 38 | P | .88 | 6.22 | R | _ | _ | _ | .96 | 6.79 | R | .04 | .28 | A |
| 39 | P | .88 | 6.22 | R | -.28 | -1.98 | L | .16 | 1.13 | A | .32 | 2.26 | R |
| 40 | P | _ | _ | _ | _ | _ | _ | -.04 | -.28 | A | _ | _ | _ |
| 41 | P | _ | _ | _ | _ | _ | _ | -.40 | -2.83 | L | _ | _ | _ |
| 42 | P | .20 | .89 | A | .60 | 2.68 | R | -1.00 | -1.00 | A | .25 | 1.22 | A |
| 43 | W | .16 | .69 | A | .12 | .85 | A | .28 | 1.98 | R | -.60 | -4.24 | L |
| 44 | W | _ | _ | _ | .32 | 2.26 | R | _ | _ | _ | -.11 | -.33 | A |
| 45 | W | -.20 | -1.41 | A | _ | _ | _ | -.08 | -.57 | A | _ | _ | _ |
| 46 | W | .76 | 5.37 | R | .00 | .00 | A | .76 | 5.37 | R | .38 | 2.47 | R |
| 47 | W | -.28 | -1.98 | L | .27 | .90 | A | -.52 | -3.68 | L | _ | _ | _ |
| 48 | W | 1.00 | 2.45 | R | _ | _ | _ | .00 | .00 | A | _ | _ | _ |
| 49 | W | .04 | .28 | A | _ | _ | _ | .08 | .57 | A | _ | _ | _ |
| 50 | W | -.04 | -.28 | A | .00 | .00 | A | -.04 | -.28 | A | _ | _ | _ |
| 51 | W | -.16 | -1.13 | A | _ | _ | _ | .08 | .57 | A | _ | _ | _ |
| 52 | W | .04 | .28 | A | _ | _ | _ | -.04 | -.28 | A | _ | _ | _ |
| 53 | W | -.40 | -2.83 | L | -.28 | -1.98 | L | -.12 | -.85 | A | -.44 | -3.11 | L |
| 54 | W | .08 | .57 | A | .70 | 3.66 | R | .68 | 4.81 | R | -1.00 | -3.61 | L |
| 55 | W | -.52 | -3.68 | L | .12 | .85 | R | -.32 | -2.26 | L | _ | _ | _ |
| 56 | W | -.36 | -2.55 | L | .28 | 1.98 | L | -.68 | -4.81 | L | _ | _ | _ |
| 57 | W | .69 | 2.50 | R | -1.00 | -1.00 | A | 1.00 | 2.00 | R | .00 | .00 | A |
| 58 | W | _ | _ | _ | _ | _ | _ | -.54 | -1.94 | L | _ | _ | _ |
| 59 | W | .56 | 2.36 | R | _ | _ | _ | -.33 | .00 | A | _ | _ | _ |
| 60 | W | _ | _ | _ | _ | _ | _ | .60 | 1.34 | A | _ | _ | _ |
